# Massive Leiomyomatous Uterine Proliferation Following Kidney Transplantation: A Case Report and Literature Review

**DOI:** 10.1155/2018/3874937

**Published:** 2018-11-28

**Authors:** Emmanuel J. Minja, Miguel Tan, Melissa J. Gibbs, Marwan M. Kazimi, Jonathan C. Hundley, Harrison S. Pollinger

**Affiliations:** Piedmont Hospital Atlanta, Piedmont Transplant Institute, 1968 Peachtree Street NW, 77 Building, 6 Floor, Atlanta, GA 30309, USA

## Abstract

Uterine fibroids are the most common benign uterine tumors affecting > 50% of premenopausal women. The incidence, burden and symptoms from uterine fibroids are higher in women of African descent compared to Caucasians. Despite increasing number of African American females being evaluated for and undergoing kidney transplantation (KT), perioperative management guidelines for uterine fibroids currently do not exist. We present a case of a 40 y/o African American female with known symptomatic uterine fibroids preoperatively and medically managed, who underwent a successful KT and 4 years later progressively developed massive leiomyomatous uterine proliferation, causing a complete lateral displacement of the transplanted kidney with severe hydronephrosis, transplant ureteral obstruction and secondary urinary tract infections with bacteremia. This obstruction required a percutaneous nephrostomy tube placement followed by an interval transabdominal hysterectomy, which was complicated by transplant ureteral transection requiring ureteral reimplantation, resulting in prolonged hospitalization, follow-up and outpatient antibiotic regimen. There is a need for management guidelines for uterine fibroids incidentally encountered during the KT evaluation process to avoid similar preventable post-KT complications in patient populations most commonly affected. Literature review and perioperative management/surveillance strategies are provided.

## 1. Case Report

Uterine fibroids are the most common benign uterine tumors affecting > 50% of premenopausal women [1]. The incidence, burden and symptoms from uterine fibroids are higher in women of African descent compared to Caucasians [2, 3]. Many African Americans females (AAFs) are also increasingly being evaluated for KT for definitive management of end-stage renal disease (ESRD) [4]. To date, however, there are no kidney transplant (KT) perioperative management guidelines for uterine fibroids, especially in this patient population that is most commonly affected. Although native ureteral obstruction secondary to uterine fibroids has been well described in literature [5, 6], three publications exist, one in Taiwan [7] and two in Japan [8, 9], describing KT ureteral obstruction from uterine fibroids.

We present a 40 y/o AAF with history of ESRD secondary to type 2 diabetes and hypertension. She was on peritoneal dialysis (PD) uneventfully for 4 years. She underwent our institutional standard preoperative evaluation for KT candidacy. Her medical history was also significant for menorrhagia secondary to uterine fibroids, managed by her gynecologist symptomatically. Her preoperative evaluation included an abdomen/pelvis computer tomography (CT) scan to assess iliac vessels' quality. No vascular contraindications for KT were identified. Noted, however, was an enlarged leiomyomatous uterus (~10x12cm cranial-caudally), but it was deemed not surgically prohibitive. Seven months prior, she had a routine surveillance pelvic ultrasound reportedly showing an enlarged uterus (10.3 x 14.0 x 14.3 cm).

On 12/3/2012 she received an excellent KT offer from a 17 y/o deceased donor with normal renal function. She underwent a standard deceased donor KT with right retroperitoneal approach and vascular anastomosis to external iliac vessels, ureteralneocystostomy with stent placement and PD catheter removal. She received standard induction immunosuppression (IS) with Thymoglobulin and maintenance IS using Tacrolimus, Mycophenolate mofetil and a prednisone taper. She received a standard infectious disease prophylaxis using a combination of Valganciclovir and Sulfamethoxazole/Trimethoprim. Postoperatively she was continued on antihypertensives and insulin therapy. She was discharged home on postoperative day (POD)#4 with excellent urine output and serum creatinine (Cr) of 0.8 mg/dL.

She was readmitted on POD#15 for abdominal pain, fever and leukocytosis. Her workup revealed acalculous cholecystitis for which she underwent laparoscopic cholecystectomy. She was discharged home 3 days later with a serum Cr of 0.63 mg/dL. As part of her work up on this admission, she underwent an abdomen/pelvis CT scan, which again demonstrating an enlarged leiomyomatous uterus, similar in size compared to CT scan 1-year prior.

**Figure 1:**
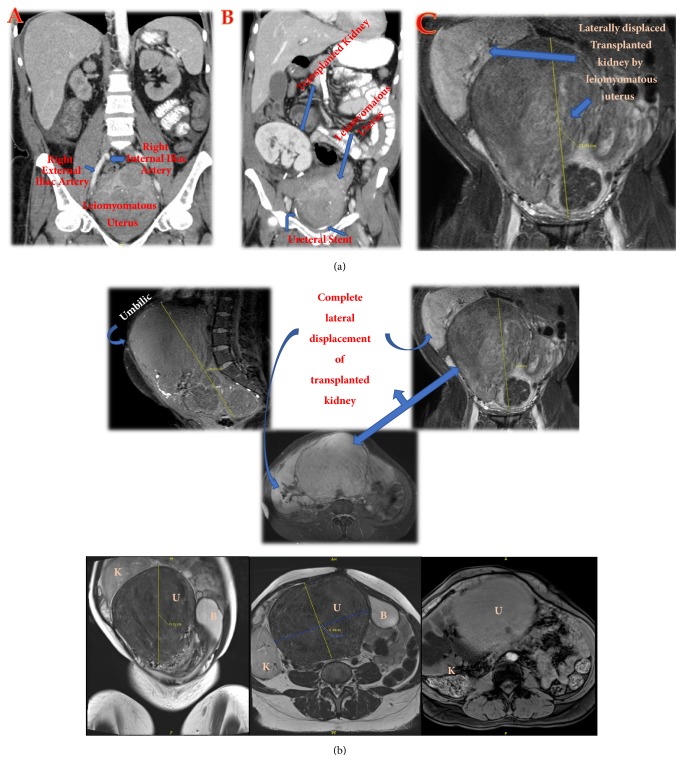
(a) Abdomen/pelvis CT scan and MRI showing progressive enlargement of the leiomyomatous uterus with time: pretransplant (A-12.2cms), immediately posttransplant (B-12.2 cms), and 4 years after transplant (C– 21.6 cms). Note: A=8/11/2012, B=12/12/2012, and C=11/01/2017. (b) Abdomen/pelvis MRI depiction of massively enlarged leiomyomatous uterus (U) completely replacing the urinary bladder (B) and transplanted kidney (K), causing severe hydronephrosis. Note that the uterus fundus is extending 5 cms above the umbilicus.

Subsequent postoperative course was also complicated by BK viruria, which resolved following reduction of IS dosage. She continued to have excellent renal function with serum Cr 0.6-1.0 mg/dL and resumed postoperative care per protocol. In the following years she continued with gynecologic follow up, as she was still having symptomatic menorrhagia despite Epoetin alfa administration and birth control pills. She continued to have excellent renal graft function. Due to these persistent symptoms however, she underwent a pelvic magnetic resonance imaging (MRI) to evaluate her symptomatic menorrhagia, now ~ 4-years post-KT, surprisingly revealing a massively enlarged uterus causing complete lateral displacement of transplanted kidney with severe hydronephrosis (Figures 1(a) and 1(b)).

A total abdominal hysterectomy (TAH) was planned for definitive management but her surgery was postponed with concerns for wound complications given her poor glycemic control at the time (HgA1c > 9). Her Cr remained unchanged (< 1.0 mg/dL). In 9/2017, she presented to an outside hospital with nausea and vomiting for 1 day, and 1-week history of missed transplant medications due to insurance coverage lapse. Her creatinine on that admission was 1.4 mg/dL, which rapidly increased to 2.06 mg/dL. Her urinalysis was consistent with a urinary tract infection (UTI). She progressed to having Escherichia coli (*E. coli*) UTI bacteremia, for which she was started on Piperacillin/Tazobactam then switched to Meropenem following an Extended Spectrum Beta Lactamase (ESBL)* E. coli* diagnosis.

A percutaneous nephrostomy tube (PNT) was immediately placed by intervention radiology (IR) for sepsis management. The Mycophenolate mofetil dose was briefly reduced during this septic shock episode. The PNT was eventually internalized to a double J stent (8.5F x 24cm) traversing from transplant renal pelvis into the bladder. The patient was kept of intravenous (IV) antibiotics, while the PNT was kept to gravity awaiting TAH, following resolution of bacteremia. Her Cr subsequently decreased to 0.99 mg/dL.

On 11/10/2017 she underwent a robotically assisted TAH by gynecology service, which was converted to an open hysterectomy due to intraoperative transplant ureter injury. The transplant ureter was inadvertently transected at level of the bladder due to the sheer size of the uterus. The transplant ureteral was reimplanted to the bladder over the previously internalized PNT. Final pathology was benign multiple leiomyomata with degenerative changes, measuring 20.5 cms in largest dimensions. She was discharged home on POD#3 doing well with stable Cr. She had one subsequent hospital readmission for ESBL* E. coli* UTI. She continued IV antibiotics on discharge and her PNT was kept open to gravity. She underwent a successful ureteral stent exchange and ureteroplasty due to ureteral irregularity noted on a pullback nephrostogram which subsequently (one month later) resolved, with prompt drainage of contrast into the bladder without a stent. PNT was finally removed. Her current Cr is < 1.0 mg/dL and she is currently back to working fulltime.

## 2. Discussion

Uterine fibroids are the most common benign uterine tumors affecting > 50% of premenopausal women and are the leading causes of hysterectomies in this cohort of patients [1]. Women of African descent have a much higher incidence of uterine fibroids [10–12] and tend to be more symptomatic, causing anemia and infertility [3]. Treatment modalities for these fibroids vary widely from birth control medications to minimally invasive interventions such as uterine artery embolization (UAE) to more invasive procedures such as hysteroscopically/laparoscopically/robotically assisted myomectomies/hysterectomies to radical open total abdominal hysterectomies (TAH) [12–14]. The success rates of these treatment modalities vary widely also.

What is also very common is the incidence of chronic kidney disease (CKD)/ESRD in African Americans, with increasing number of patients being evaluated for KT [4]. According to US Organ Procurement and Transplantation Network (OPTN) data for kidney transplants performed in 2017, of the 19,850 KT recipients, 2075 (10.45%) were AAFs, of which 873 (42%) were between 18-49 years of age [15]. Currently there are >5100 actively listed AAFs in this same age group awaiting KT nationwide. Postulating from data on prevalence of symptomatic fibroids, potentially > 400 AAFs who received kidney transplants in 2017, and currently > 2500 AAFs who are currently actively listed waiting for kidney transplants, could potentially have symptomatic uterine fibroids.

Chen et al. [7] described four successful laparoscopically assisted transvaginal hysterectomies following KT in Taiwan in premenopausal women with symptomatic uterine fibroids, weighing between 160-380 grams, demonstrating the safety of this surgical intervention. Relative to our patient, these sizes are miniscule. Takewaza et al. [8] described a simple hysterectomy performed in Japan for alleviation of ureteral obstruction from uterine fibroid. Hara et al. [9] described also a similar KT ureteral obstruction by uterine fibroid for a patient in Japan that was monitored by ultrasonography because of unchanged serum creatinine. What is interesting is the lack of publications of this entity in patients who are most commonly affected, that is, women of African descent.

Our case report demonstrates that significant leiomyoma-related postoperative morbidity can occur after KT. The amount of leiomyomatous proliferation in our case was significant and prominent. In retrospect, recommending uterine artery embolization (UAE) pretransplant would have been very beneficial for this patient, as studies have shown this procedure carries > 90% success rates in reducing the sizes of, and pain from, fibroids and menorrhagia [16]. Care needs to be taken, however, during the approach for UAE because the main access to the internal iliac artery for embolization is via the contralateral femoral artery and external iliac artery. Careless instrumentation can make the external iliac arterial exposure during KT more difficult secondary to periarterial inflammation. This procedure however could dramatically reduce the possible post-KT complications from proliferation of the leiomyomatous uterus, especially in premenopausal women.

We suggest premenopausal women with notably enlarged symptomatic uterine fibroids who do not undergo such interventions pre-KT, should be followed with serial pelvic ultrasounds every 6 months to monitor for potential growth of the uterus and most importantly, understand its relationship to the transplanted kidney before becoming pathologically large. If there is a rapid increase in size, a CT scan can be performed for further delineation of anatomy and facilitate surgical intervention planning.

Rapidly enlarging leiomyomatous uteri in postmenopausal women should be aggressively evaluated for possible sarcomatous transformation [17, 18] and appropriate actions taken early. Alternatively, for symptomatic patients (pain, anemia or menorrhagia) with no further interest in child bearing, and certainly for postmenopausal women, minimally invasive hysterectomy should be pursued with specific goal to avoid violation of the retroperitoneal space, preserved for KT exploration.

It was unclear why our patient's fibroids proliferated with such rapidity post-KT. Whether transplant immunosuppressants caused proliferation of these benign tumors in a manner similar to their effects on other malignant tumors [19, 20] needs further elucidation. We hope the transplant community would share specific experiences regarding uterine fibroids management to help create universal guidelines pre- and post-KT to avoid similar potential morbidity associated with this entity.

In summary, AAF patients with CKD/ESRD with symptomatic uterine fibroids should undergo careful KT evaluation. Premenopausal patients with large and symptomatic fibroids should consider UAE, pre-KT, with careful attention paid to external iliac arteries' instrumentation. Premenopausal symptomatic patients with no child-bearing aspirations and certainly postmenopausal patients, with large leiomyomatous uteri that surgically limit access to iliac vessels and urinary bladder, should be evaluated for laparoscopic/robotic hysterectomy pre-KT with complete preservation of the retroperitoneal space. Alternatively, patients with enlarged uteri that are not surgically prohibitive for KT should have serial pelvic ultrasounds post-KT to monitor for growth of the leiomyomatous uterus in relation to the transplanted kidney. If there's significant growth in a short span, an abdomen/pelvis CT should be obtained to further delineate clear anatomy and facilitate appropriate interventions before the fibroids proliferate to a pathologic size.

## Abbreviations

AAF: African American female
CKD: Chronic kidney disease
Cr: Creatinine
CT: Computer tomography
ESRD: End-stage renal disease
*E. coli*: Escherichia coli
ESBL: Extended spectrum beta lactamase
IS: Immunosuppression
IR: Intervention radiology
IV: Intravenous
KT: Kidney transplantation
MRI: Magnetic resonance imaging
OPTN: Organ procurement and transplant network
PNT: Percutaneous nephrostomy tube
PD: Peritoneal dialysis
POD: Postoperative day
TAH: Total abdominal hysterectomy
UTI: Urinary tract infection
UAE: Uterine artery embolization.
